# SODNet: a scale-oriented detection network for efficient UAV-based sewage outfall detection

**DOI:** 10.1038/s41598-026-45595-x

**Published:** 2026-03-26

**Authors:** Luping Zeng, Xiaozhou Liu, Bingyang Dai, Liangming Wen, Zhiguo Du

**Affiliations:** Business College, Southwest University, Chongqing, 402460 China

**Keywords:** Sewage outfalls, Object detection, YOLOv8, Multi-scale fusion, Lightweight, Engineering, Environmental sciences, Mathematics and computing

## Abstract

Accurate identification of river sewage outfalls is crucial for effective water pollution control. Unmanned Aerial Vehicles (UAVs), with their high mobility and wide coverage, have become a vital tool for this monitoring task. However, this application is hampered by the dual challenges of robust multi-scale object detection and lightweight model deployment on computationally limited platforms. To address the trade-off between accuracy and efficiency, this study proposes an efficient deep learning-based detection method, termed Scale-Oriented Detection Network (SODNet). Specifically, we propose an Efficient Context Feature Pyramid Network (ECFPN) to enhance multi-scale feature representation. Additionally, a shared decoupled head with a Multi-Scale Grouped Fusion (MSGF) module strengthens feature extraction while reducing computational costs. Furthermore, a channel pruning strategy is employed to compress the model, notably improving inference speed. Experimental results demonstrate that SODNet achieves an AP@50 of 89.9% and a precision of 91.1%, representing improvements of 1.2% and 2.7% over the baseline model, respectively. Meanwhile, parameters and GFLOPs are reduced by 77.5% and 73.6%. On a deployed edge device, SODNet achieves 40.3 FPS. These findings indicate that SODNet gains substantial computational efficiency while maintaining excellent detection performance, making it ideal for resource-constrained UAV scenarios and offering a feasible solution for intelligent environmental supervision.

## Introduction

Water pollution, exacerbated by rapid economic growth and accelerating urbanization, has become an increasingly severe issue^1^. Sewage outfalls, as direct conduits for pollutants, persistently degrade water quality and threaten ecosystem health^2–4^. Recent studies have further highlighted the profound environmental implications of inadequate sewage outfall monitoring^5^, noting that unmanaged discharges and accidental spills can cause cascading ecological damage across both inland and broader marine environments^6^. To address this challenge, the routine and detailed monitoring of these outfalls is imperative for effective regulation. However, traditional manual inspection methods are labor-intensive, inefficient, and prone to omissions, rendering them inadequate for high-frequency, large-scale monitoring^7^. In this context, Unmanned Aerial Vehicle (UAV) remote sensing presents a promising alternative, offering high mobility, wide coverage, and high-resolution imaging^8,9^. Nevertheless, conventional workflows still depend on manual visual interpretation of UAV imagery, a process that is time-consuming, subjective, and difficult to scale^10^.

In recent years, deep learning techniques, particularly Convolutional Neural Networks, have achieved groundbreaking progress in computer vision due to their superior feature learning and pattern recognition capabilities^11–13^. Object detection methods based on deep learning have substantially outperformed traditional methods, establishing themselves as the mainstream solution for complex visual recognition tasks^14,15^. However, their application to the automated detection of sewage outfalls in UAV imagery still faces several distinct challenges.

First, sewage outfalls exhibit significant scale variations in UAV imagery captured from different altitudes and viewing angles. While large outfalls present clear visual features, smaller or partially concealed ones are often obscured by complex backgrounds, such as riverbanks, vegetation, or shadows, resulting in weak, ambiguous visual cues. This multi-scale characteristic places stringent demands on the feature representation capacity of detection models^16^. Traditional architectures often struggle to effectively represent features across a single layer, leading to missed detections of small targets and inaccurate localization of large ones^17,18^.

Second, UAVs are resource-constrained edge computing platforms, with onboard processors that have strict limitations on computational power, memory, and energy consumption. Therefore, deploying deep learning models with numerous parameters and high computational complexity for real-time monitoring is often infeasible^19,20^. Although many high-accuracy detection models perform exceptionally well on server-side systems, their inference speeds are typically insufficient for the real-time demands of dynamic UAV patrols, limiting their practical utility^21^. Consequently, lightweight model design has become a critical challenge for efficient deployment. This requires reducing parameters and computational cost while enhancing inference speed, without compromising detection accuracy^22^.

Motivated by the dual challenges of multi-scale detection and lightweight deployment, we propose the Scale-Oriented Detection Network (SODNet), a lightweight model engineered explicitly for UAV-based sewage outfall detection. The SODNet architecture first employs a backbone network to extract multi-level features. Subsequently, the Efficient Context Feature Pyramid Network (ECFPN) is integrated into the neck to enhance contextual awareness and feature fusion across different scales. The detection head then utilizes a shared decoupled design combined with the Multi-Scale Grouped Fusion (MSGF) module to strengthen multi-scale feature representation while minimizing computational redundancy. Finally, we apply a channel pruning strategy to the optimized model for deep compression, further satisfying the requirements for lightweight deployment. Our main contributions are fourfold:

1. Propose the ECFPN, consisting of a context-awareness module and a feature fusion module, to enhance the responsiveness of multi-scale features to occluded targets while effectively suppressing background interference.
2. Introduce the Adaptive Context Feature Fusion (ACFF) mechanism, which integrates Context Anchor Attention (CAA) with the Efficient Upsampling Convolution Block (EUCB) to achieve adaptive fusion of high- and low-level features.
3. Incorporate a shared decoupled head and design the MSGF module to improve multi-scale feature representation while reducing computational cost. Combined with the MPDIoU loss function, detection accuracy is further enhanced by jointly considering bounding box positions and scale differences.
4. Adopt a channel pruning strategy to remove redundant channels, significantly compressing model size while accelerating inference speed, thereby improving adaptability to resource-constrained platforms.

## Related work

### Object detection algorithms for sewage outfall detection

In sewage outfall detection tasks, targets are typically situated in complex scenarios and may be occluded. UAVs must process a large number of images in a short time to achieve real-time detection. Consequently, real-time performance, detection accuracy, and lightweight design are critical for object detection algorithms. At present, object detection methods are typically classified into two primary types: two-stage and one-stage frameworks. In the two-stage paradigm, typified by Faster R-CNN^23^, the detector first proposes candidate regions and subsequently performs classification and localization. The one-stage paradigm takes a different approach, with models such as You Only Look Once (YOLO)^24^ producing bounding box and category predictions directly from the input image in a single step. Although two-stage methods generally offer higher accuracy, they suffer from lower real-time performance and higher computational demands.

Previous studies have also used two-stage approaches to detect sewage outfalls. For example, Huang et al.^22^ proposed an Attention-Based Feature Enhancement Network to detect sewage spills from low-altitude UAV imagery. This two-stage algorithm enhances feature representation through a Global Context Block and an RoI Attention Module, achieving an accuracy of 72.5%. However, it requires up to 55.10 million parameters and 481.64 GFLOPs. In another study, Huang et al.^25^ improved the Faster R-CNN framework by introducing a geospatial deep learning approach to optimize the recognition of sewage outfalls. Yet, they did not address the impact of model size. Overall, although two-stage methods offer higher detection accuracy, their large parameters and high computational cost impose stringent requirements on deployment platforms. In contrast, one-stage methods can achieve competitive accuracy with fewer parameters and efficient computation, making them more suitable for lightweight scenarios^26^. For instance, recent research has explored lightweight one-stage architectures, such as an improved YOLOv10n model that integrates spatial-depth conversion, convolution, and attention mechanisms to detect small, severely occluded sewage outfalls efficiently^27^. One objective of this study is to construct a lightweight model to alleviate the excessive parameter and computational burdens. With this consideration in mind, we focus on lightweight solutions tailored for sewage outfall detection and adopt YOLOv8 as the baseline model, given its stable performance and broad applicability among one-stage methods.

### Multi-scale feature fusion

Images of sewage outfalls captured by UAVs from varying altitudes and viewing angles exhibit significant scale variations. This places stringent demands on a model’s multi-scale detection capability, a challenge that is particularly acute for small targets characterized by sparse features. Conventional detectors address this by integrating features from multiple network layers, typically in the neck of the architecture. Mainstream approaches, such as the Feature Pyramid Network (FPN) and Path Aggregation Network (PAN)^28–30^, primarily rely on element-wise addition for feature fusion. However, these methods suffer from two key limitations. First, they lack a context-aware enhancement mechanism, which restricts the optimization and representational power of the fused features. Second, when processing targets of disparate scales, their reliance on simple addition fails to harmonize semantic information across layers effectively.

To overcome these issues, several advanced methods have been proposed. For instance, Cai et al.^31^ developed PKINet for remote sensing imagery, which uses non-dilated multi-scale convolution kernels to extract features and capture local context. Qiu et al.^32^ introduced a Multi-scale Binocular Pixel-Adaptive Feature Fusion Module that works in conjunction with a Cross-Scale Feature Aggregation Module to achieve dynamic, dual-path multi-scale feature fusion. Chen et al.^33^ proposed the Hierarchical Scale-based Feature Pyramid Network, which employs feature selection and selective fusion mechanisms to address multi-scale detection challenges in white blood cells effectively. Despite these advances, a common drawback of these methods is their substantial computational complexity. This complexity hinders their efficient deployment on resource-constrained platforms, which are constrained by strict limits on computational power and memory.

### Lightweight network design

Limited computational capacity, storage resources, and power consumption typically constrain UAV platforms. Lightweight models address this issue by optimizing network structures to balance performance and resource consumption. Existing studies have explored different directions: Feng et al.^34^ introduced Hyper-YOLO, which leverages hypergraph computation to model complex high-order correlations among visual features; Rahman et al.^35^ designed a multi-scale convolutional attention decoder that employs multi-scale depth convolutional blocks, achieving state-of-the-art performance in multiple medical image segmentation tasks with a remarkably low number of parameters; Huang et al.^36^ adopted a lightweight YOLOv11 model for online multi-camera multi-vehicle tracking, achieving improved tracking accuracy in complex environments while ensuring deployability on edge devices. However, the aforementioned approaches, while employing lightweight designs to achieve some efficiency, have not explored network pruning to enhance model compactness further.

Network pruning, as an effective model compression technique, can significantly reduce computational overhead and memory usage while maintaining accuracy, making it particularly suitable for resource-constrained platforms^37^. Zhong et al.^38^ applied Fisher pruning to compress the SiamFC++ model for UAV tracking, achieving a favorable balance between accuracy and efficiency; Choi et al.^39^ proposed a channel importance metric combining detection-related saliency and position awareness, explicitly designed for autonomous driving detectors, which reduced model size and computation with negligible accuracy loss; Wu et al.^40^ employed channel pruning on YOLOv4 to enable efficient and rapid detection of apple blossoms, significantly reducing parameters and computation while preserving accuracy. These findings suggest that integrating network pruning with lightweight model design can substantially improve efficiency and provide a solid foundation for practical deployment on UAV platforms.

## Methodology

**Fig. 1:**
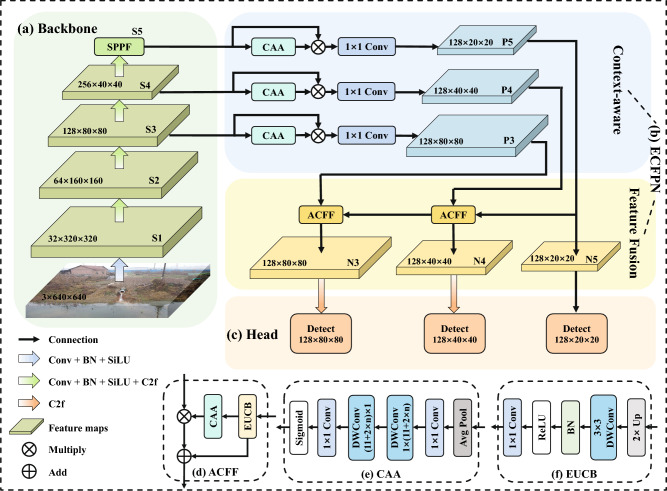
Overall architecture of SODNet and detailed designs of key modules. (**a**) Backbone network; (**b**) Efficient Context-aware Feature Pyramid Network (ECFPN) neck; (**c**) Detection head; (**d**) Adaptive Context Feature Fusion (ACFF); (**e**) Context Anchor Attention (CAA); (**f**) Efficient Upsampling Convolution Block (EUCB).

We introduce SODNet, a detection model that incorporates scale-oriented design and lightweight deployment. SODNet consists of three components: Backbone, Neck, and Head. The backbone progressively extracts semantic information from shallow to deep layers while maintaining a relatively high level of detail resolution. The neck employs a multi-scale strategy to enable contextual interaction and cross-layer fusion of feature maps from different depths, thereby improving the network’s adaptability to scale variations. The lightweight detection head performs target localization using fused features and outputs positional information for sewage outfalls. The overall architecture and detailed design of several modules in SODNet are illustrated in Fig. 1.

Building upon the YOLOv8 framework, SODNet introduces targeted optimizations: (1) a redesigned neck network with the proposed ECFPN; (2) a lightweight shared decoupled head integrated with the MSGF module; (3) replacement of the CIoU loss function with MPDIoU; (4) channel pruning applied to the newly designed model, which further reduces parameter and computational cost while preserving high accuracy.

**Fig. 2:**
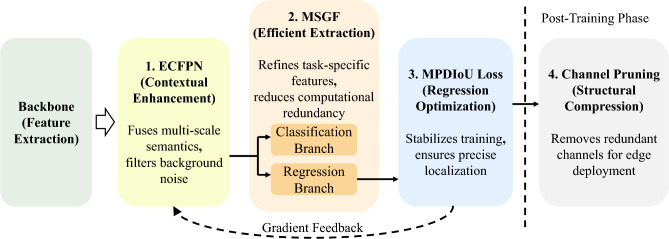
Simplified schematic of the collaborative mechanism and data flow in SODNet.

To clarify the collaborative mechanism among these targeted optimizations, Fig. 2 presents a simplified schematic illustrating their functional roles and the overall data flow. Rather than operating as isolated components, these modules synergistically form a cohesive pipeline. Specifically, the ECFPN first filters background noise and enriches multi-scale semantics. These refined features are then fed into the shared decoupled head, where the MSGF module extracts task-specific representations while actively suppressing computational redundancy. During the training phase, the MPDIoU loss strictly supervises this process by stabilizing bounding box regression. Finally, once the network has learned optimal feature representations, the channel pruning strategy evaluates and removes redundant connections.

### Efficient context feature pyramid network

To articulate the motivation, Table 1 summarizes the architectural differences between our design and existing multi-scale fusion paradigms. In UAV-based outfall detection, traditional FPN-PAN models treat multi-scale features equally, limiting their ability to suppress background noise. Advanced designs, such as the Bidirectional Feature Pyramid Network (BiFPN)^30^, introduce weighted cross-scale connections but lack explicit contextual filtering mechanisms. While High-Level Screening-feature Fusion Pyramid Network (HSFPN)^33^ utilizes high-level semantics to guide low-level features, its reliance on complex multi-level attention operations can lead to sub-optimal parameter distribution. ECFPN is specifically designed to filter complex background interference and enhance precision. By integrating CAA and ACFF, ECFPN achieves context-aware filtering while streamlining fusion paths, ensuring a highly rational balance between detection precision and computational efficiency.

**Table 1:** Architectural differences and theoretical comparison of multi-scale fusion modules.

Method	Core mechanism	Architectural differences	Proposed improvements in ECFPN
FPN-PAN	Standard bidirectional top-down and bottom-up feature fusion	Fuses features via simple element-wise addition without assessing spatial importance, making it vulnerable to background noise	ECFPN introduces context-aware filtering before fusion to actively suppress background interference and improve precision
BiFPN	Weighted bidirectional cross-scale connections	Relies on scalar weights for fusion but lacks explicit spatial context modeling, limiting its capability to locate ambiguous small outfalls	ECFPN uses spatial and channel-wise contextual attention rather than simple scalar weights, capturing long-range dependencies for better target localization
HSFPN	Uses high-level features to filter low-level ones via attention	Complex multi-level attention operations introduce redundant computational overhead during feature alignment	ECFPN streamlines the fusion pathway via the EUCB and ACFF, minimizing GFLOPs while maintaining strong semantic guidance
ECFPN (ours)	Context-aware adaptive filtering and feature fusion	Specifically designed for UAV edge deployment with strict computational constraints	Achieves the optimal balance by maximizing detection precision and maintaining the lowest computational cost

Guided by these design principles, the architecture of the proposed ECFPN is illustrated in Fig. 1b. It tackles the multi-scale challenges through two synergistic components: the context-aware module and the feature fusion module.

**Context-aware module:** The CAA module (Fig. 1e) is a key component of this process. It takes the input feature map $f_{\text {in}} \in \mathbb {R}^{C \times H \times W}$, where *C* is the number of channels, *H* the height, and *W* the width. Specifically, the CAA module first applies global average pooling to $f_{in}$ to extract global contextual information and then employs a $1 \times 1$ convolution to capture local region features. Subsequently, two depth-wise strip convolutions are introduced along the horizontal and vertical directions to establish long-range dependencies. This convolutional strategy enlarges the receptive field while mitigating computational overhead, resulting in a substantial reduction in parameters relative to conventional large-kernel designs. After processing with the depth-wise strip convolutions, the feature map passes through a $1 \times 1$ convolution followed by the Sigmoid function to generate an attention weight map $f_{\text {CAA}} \in \mathbb {R}^{C \times 1 \times 1}$. These weights dynamically adjust feature intensities across spatial positions, highlighting regions associated with sewage outfalls while suppressing background noise. Finally, $f_{\text {CAA}}$ is multiplied with $f_{\text {in}}$ channel by channel to obtain an enhanced feature representation. A $1 \times 1$ convolution reduces the channels to 256, thereby maintaining uniform feature dimensions across scales. This facilitates effective fusion while reducing computational cost and provides a solid foundation for subsequent fusion.

**Fig. 3:**
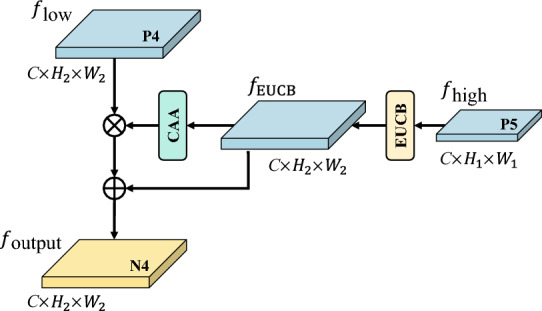
ACFF mechanism.

**Feature fusion module:** We propose the ACFF mechanism that leverages high-level semantic information to guide the fusion of low-level features. The detailed structure is shown in Fig. 3. Traditional pixel-wise summation methods assign equal weights to all pixels, ignoring spatial relationships and contextual dependencies, and thus cannot dynamically adjust weights based on feature importance. By contrast, ACFF adaptively adjusts the fusion weights of high- and low-level features according to contextual information, thereby improving the discriminability and robustness of multi-scale features. The high-level feature $f_{\text {high}} \in \mathbb {R}^{C \times H_1 \times W_1}$ is first upsampled through the EUCB module (Fig. 1f) so that its output matches the size of the low-level feature $f_{\text {low}} \in \mathbb {R}^{C \times H_2 \times W_2}.$ The operation is defined as:

1 $$\begin{aligned} f_{\text {EUCB}} = Conv_{1 \times 1}\Big ( ReLU\big ( BN\big ( DWConv(Up(f_\text {high})) \big ) \big ) \Big ) \end{aligned}$$

where $Conv_{1 \times 1}$ represents a $1 \times 1$ convolution, *ReLU* is the activation function, *BN* denotes batch normalization, *DWConv* represents depth-wise convolution, and *Up* indicates upsampling with a scale factor of 2. The resulting feature map is $f_{\text {EUCB}} \in \mathbb {R}^{C \times H_2 \times W_2}$. Next, $f_{\text {EUCB}}$ is passed into CAA to generate an adaptive attention weight map:

2 $$\begin{aligned} W_{\text {CAA}} = CAA(f_{\text {EUCB}}) \end{aligned}$$

Finally, the context-guided fusion of low- and high-level features produces the enhanced feature map:

3 $$\begin{aligned} f_{\text {output}} = f_{\text {low}} \cdot W_{\text {CAA}} + f_{\text {EUCB}} \end{aligned}$$

ECFPN demonstrates significant advantages in sewage outfall detection tasks. CAA captures long-range spatial dependencies and integrates both global and local contextual information, allowing the model to accurately highlight outfall regions under complex backgrounds–particularly for detecting small or irregularly shaped targets. Meanwhile, ACFF adaptively fuses high- and low-level features under contextual guidance: high-level features contribute rich semantic information, while low-level features preserve fine-grained boundaries. This enables the model to maintain stable performance when detecting outfalls of varying scales.

### Shared decoupled head with MSGF module

**Fig. 4:**
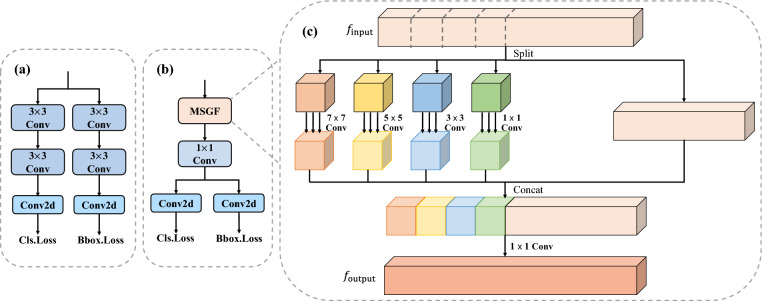
Comparison of head optimization. (**a**) Baseline decoupled head. (**b**) Proposed decoupled head. (**c**) MSGF module.

Existing object detection frameworks typically adopt a decoupled head design, where bounding box regression and category classification are processed independently. This design reduces interference between tasks, allowing each task to focus more effectively on its computation, thereby significantly improving detection accuracy^41^. However, it also introduces computational redundancy and parameter overhead, which increases the computational and storage burden. A novel detection head architecture is designed to address this problem, as depicted in Fig. 4b. This structure retains the advantages of the decoupled design while incorporating a mechanism that shares convolution across different tasks and integrates a lightweight multi-scale convolution module, MSGF. The MSGF module effectively enhances feature extraction capacity, particularly for multi-scale information, while reducing computational and parameter costs. Its design is illustrated in Fig. 4c.

The MSGF module is specifically designed to enhance the extraction and fusion of multi-scale information. The overall process is as follows: the original feature map is first divided into two parts. One part preserves the original feature information, while the other is further split into four equally sized subregions:

4 $$\begin{aligned} f_{\text {input}} = \left\{ {f_{\text {half}}, f_{\text {split}}^{(1)}, f_{\text {split}}^{(2)}, f_{\text {split}}^{(3)}, f_{\text {split}}^{(4)}} \right\} \end{aligned}$$

This partitioning preserves global semantic information while enabling the model to focus on local regions, thereby enhancing its multi-scale feature perception capability. Next, MSGF applies convolution kernels of different sizes in parallel to each split region:

5 $$\begin{aligned} f_{\text {Conv}} = \left\{ {\operatorname {Conv}_{1 \times 1}(f_{\text {split}}^{(1)}), \operatorname {Conv}_{3 \times 3}(f_{\text {split}}^{(2)}), \operatorname {Conv}_{5 \times 5}(f_{\text {split}}^{(3)}), \operatorname {Conv}_{7 \times 7}(f_{\text {split}}^{(4)})} \right\} \end{aligned}$$

Smaller kernels capture fine-grained local features, while larger kernels capture long-range contextual information. This combination improves the model’s adaptability to targets of different scales, enhancing its robustness in complex scenarios. Finally, MSGF concatenates the multi-scale convolution outputs with the preserved original features and applies a $1 \times 1$ convolution for feature compression and fusion:

6 $$\begin{aligned} f_{\text {output}} = \operatorname {Conv}_{1 \times 1}\left( \left[ f_{\text {Conv}} , f_{\text {half}} \right] \right) \end{aligned}$$

This operation not only retains the information from the original feature maps but also effectively integrates features extracted at different scales, thereby strengthening multi-scale feature fusion. Through the final $1 \times 1$ convolution, the model achieves more efficient integration of multi-scale information, improving both robustness and accuracy when handling objects of varying sizes and scales.

### MPDIoU for precise bounding box regression

**Fig. 5:**
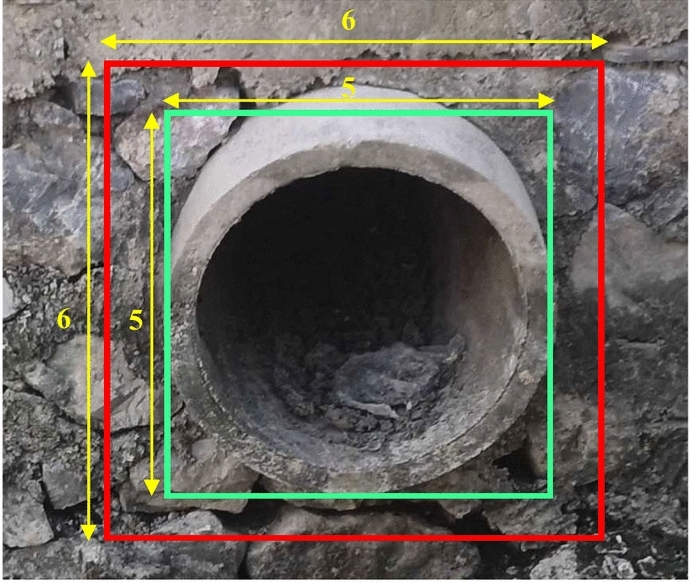
The case where the aspect ratio and the center point of the predicted box coincide with those of the ground-truth box.

In object detection tasks, bounding box regression is a key step for accurately predicting object location and size. Its core principle is to improve localization accuracy by reducing the relative gap between the predicted and ground-truth boxes. Existing models commonly adopt the CIoU loss function^42^. However, as illustrated in Fig. 5, if the predicted and ground-truth boxes have identical aspect ratios and coinciding centers, CIoU reduces to IoU, which cannot adequately reflect relative positional relations.

The MPDIoU loss^43^ is introduced to overcome this limitation. Unlike CIoU, which relies on center distance or aspect-ratio consistency, MPDIoU directly compares the coordinate differences between the top-left and bottom-right corners of the predicted and ground-truth boxes. The definition is as follows:

7 $$\begin{aligned} L_{\textrm{MPDIoU}} = 1 - \operatorname {IoU} + \frac{d_1^2}{w^2 + h^2} + \frac{d_2^2}{w^2 + h^2} \end{aligned}$$

8 $$\begin{aligned} d_{1}^{2} = \left( x_{1}^{{prd}} - x_{1}^{{gt}}\right) ^{2} + \left( y_{1}^{{prd}} - y_{1}^{{gt}}\right) ^{2} \end{aligned}$$

9 $$\begin{aligned} d_{2}^{2} = \left( x_{2}^{{prd}} - x_{2}^{{gt}}\right) ^{2} + \left( y_{2}^{{prd}} - y_{2}^{{gt}}\right) ^{2} \end{aligned}$$

where $(x_1^{prd}, y_1^{prd})$ and $(x_2^{prd}, y_2^{prd})$ represent the top-left and bottom-right coordinates of the predicted box, while $(x_1^{gt}, y_1^{gt})$ and $(x_2^{gt}, y_2^{gt})$ denote the corresponding coordinates of the ground-truth box. The term $(w^2 + h^2)$ serves as a normalization factor.

**Fig. 6:**
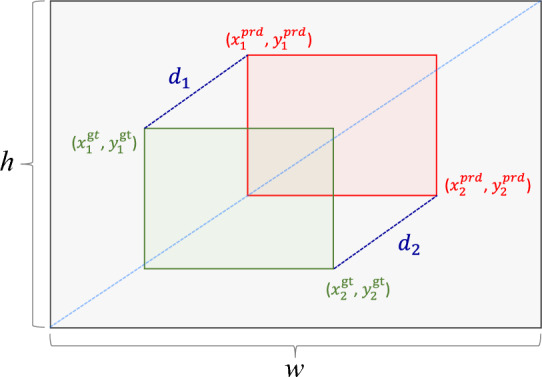
Working principle diagram of MPDIoU.

As illustrated in Fig. 6, MPDIoU explicitly computes the squared Euclidean distances between the corresponding corners of the predicted and ground-truth boxes. By directly minimizing these distances, MPDIoU provides a more precise characterization of positional relationships. The benefit is clear when both boxes share the same aspect ratio, as MPDIoU continues to capture their relative positions and enhances detection accuracy. Furthermore, by operating at the coordinate level, MPDIoU naturally encompasses the crucial metrics of existing methods, including non-overlapping areas, center distance, and aspect-ratio deviations. This direct approach also streamlines the computation.

### Channel pruning strategy

Although SODNet achieves significant compression in both parameters and computation through lightweight design, there remains room for further optimization when deploying on resource-constrained platforms. Structured pruning is a practical approach to reducing the computational cost and memory footprint of deep neural networks while preserving accuracy, making it particularly suitable for outfall detection on resource-limited platforms^44^. Based on pruning granularity, existing methods can generally be categorized into three types: weight-level, layer-level pruning, and channel-level^45^. Weight-level pruning offers fine granularity but typically requires hardware acceleration; layer-level pruning is straightforward but lacks flexibility; in contrast, channel-level pruning strikes a balance between efficiency and implementation difficulty, allowing the removal of redundant channels while maintaining the overall network architecture. Therefore, this study adopts channel pruning to reduce SODNet’s computational cost further.

**Fig. 7:**
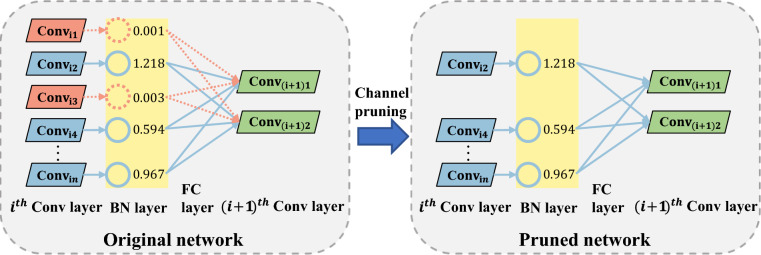
Pruning schematic diagram of channel pruning algorithm.

**Fig. 8:**
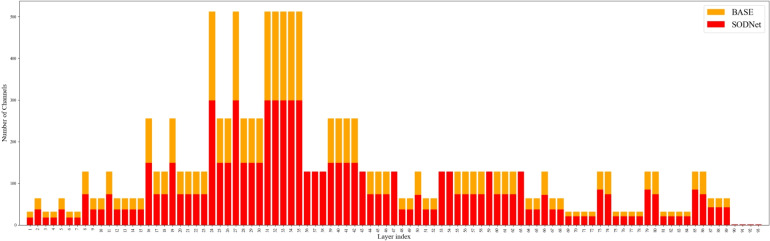
Channel changes before and after pruning.

The core principle of channel pruning is to identify and remove unimportant channels and their associated input/output connections, thereby reducing the number of parameters and computational cost. The scaling factor $\gamma$ in Batch Normalization layers can be used to assess the importance of the channel. During concatenation operations, the channels and neurons deemed irrelevant are removed. As illustrated in Fig. 7, based on the distribution of $\gamma$ and the pruning ratio, channels with higher contributions are retained, while those with less critical contributions are discarded. The channel pruning algorithm used in this study consists of the following steps:

1. Sparse training. In BN layers widely applied after convolutional layers, each channel is associated with a learnable scaling factor $\gamma$, which naturally serves as an indicator of channel importance: the larger the value, the greater the channel’s contribution to the output; conversely, smaller values indicate lower importance. The BN formulation is as follows:
  10 $$\begin{aligned} {\left\{ \begin{array}{ll} \widehat{x}_{i} = \dfrac{x_{i} - \mu _{B}}{\sqrt{\sigma _{B}^{2} + \epsilon }} \\ y_{i} = \gamma \widehat{x}_{i} + \beta \end{array}\right. } \end{aligned}$$
  where $\widehat{x}{i}$ is the normalized input, *y* *i* is the final output, $\mu _{B}$ and $\sigma _{B}^{2}$ denote the batch mean and variance, $\beta$ is the offset parameter, and $\gamma$ is the scaling factor. During training, $L_{1}$ regularization is imposed on the scaling factor $\gamma$ to promote sparsity. The corresponding loss function is:
  11 $$\begin{aligned} L = \sum _{(x,y)} l(f(x, W), y) + \lambda \sum _{\gamma \in \Gamma } |\gamma | \end{aligned}$$
  where the first term represents the standard training loss, and the second term enforces the sparsity on $\gamma$, with $\lambda$ controlling the regularization strength. This mechanism enables the model to automatically identify unimportant channels during training without introducing additional operators or parameters.
2. Channel pruning. After sparse training, the channels are ranked by the magnitude of their scaling factors $|\gamma |$. To precisely control the compression rate, an adaptive global threshold is dynamically determined based on a predefined target computational reduction ratio (i.e., the speed-up factor). Furthermore, a one-shot pruning strategy is employed. Specifically, channels with $|\gamma |$ below this adaptive threshold are considered redundant and simultaneously removed, along with their corresponding input and output connections. Following this one-shot pruning, both the model’s parameters and computational complexity are substantially reduced while preserving structural integrity. As shown in Fig. 8, the number of channels in most layers decreases significantly, demonstrating the approach’s effectiveness.
3. Fine-tuning the pruned model. Since channel removal may lead to accuracy degradation, the pruned compact model requires fine-tuning. In this stage, the network is retrained under the same task loss to restore detection accuracy.

## Results

### Experiment setup

#### Dataset

iSOOD^16^ is the first fine-grained dataset dedicated to sewage outfall detection in natural environments. The dataset focuses on sewage outfalls in the Yangtze and Yellow River Basins in China, addressing the issue that vertically captured images often overlook small outfalls. It contains 10,481 high-quality images captured by UAVs and handheld cameras, meticulously selected through a rigorous screening process that eliminates irrelevant, blurry, or low-resolution content. The dataset comprises images acquired from diverse geographic locations and under varying lighting conditions, encompassing 10 categories of sewage outfalls, including agricultural drainage, livestock wastewater, and combined sewage. This diversity provides a rich foundation for model training and testing. The dataset was randomly split into training, validation, and test sets at 8:1:1 to ensure comprehensive and representative model evaluation.

#### Performance evaluation metrics

We evaluated the performance of the proposed model using six metrics: Precision (P), Recall (R), average precision at an IoU threshold of 0.5 (AP@50), number of parameters (Params), giga floating point operations (GFLOPs) and inference speed measured in frames per second (FPS), where P, R, and AP@50 are defined as follows:

12 $$\begin{aligned} P = \frac{TP}{TP + FP} \end{aligned}$$

13 $$\begin{aligned} R = \frac{TP}{TP + FN} \end{aligned}$$

14 $$\begin{aligned} AP@50 = \int _0^1 P(R) dR \end{aligned}$$

where *TP* denotes true positives, *FP* false positives, and *FN* false negatives. *P*(*R*) represents the precision at a given recall level. Precision measures the proportion of correctly predicted positive samples among all positive predictions; higher precision indicates a lower false alarm rate. Recall measures the model’s ability to identify all ground-truth targets; higher recall corresponds to a lower miss rate. AP@50, computed by integrating precision over recall at an IoU threshold of 0.5, provides a comprehensive measure of overall detection performance.

In terms of efficiency, Params quantifies the model’s storage requirements; fewer parameters make it more suitable for deployment on resource-constrained platforms. GFLOPs measure the number of floating-point operations per forward pass, indicating computational complexity; lower GFLOPs correspond to higher computational efficiency. FPS measures the number of image frames that the model can process per second and is a critical indicator of real-time performance. Higher FPS ensures stable detection during UAV flight. We assess detection capability using accuracy-related metrics (P, R, AP@50) and efficiency using lightweight, real-time metrics (Params, GFLOPs, FPS).

#### Parameter settings

All experiments were conducted on a workstation running Ubuntu 20.04.5 LTS. The hardware platform was equipped with an Intel Xeon Platinum 8255C CPU, 40 GB of RAM, and dual NVIDIA GeForce RTX 2080 Ti GPUs (11 GB). Our implementation utilized a software stack comprising Python 3.8.10, PyTorch 2.0.0, and CUDA 11.8. For model training, we used a batch size of 32 for 200 epochs, 8 worker threads for data loading. The models were optimized using stochastic gradient descent (SGD) with an initial learning rate of 0.01. To ensure a fair and consistent comparison, all models were trained from scratch without leveraging any pre-trained weights. The FPS metric for all models was measured across the dual-GPU setup using a batch size of 32 and an input resolution of 640 $\times$ 640.

### Test results analysis

To validate SODNet’s effectiveness in outfall detection, we conducted a comprehensive evaluation against the YOLOv8 baseline on the iSOOD dataset. The results are summarized in Table 2. The experiments highlight a successful balance between performance and efficiency. Initially, the unpruned SODNet-O model reduces its complexity compared to the baseline, resulting in 40.4% fewer parameters and 33.1% less computation, while simultaneously improving precision by 2.9%, recall by 2.3%, and AP@50 by 1.4%. Subsequently, a channel pruning strategy dramatically enhances these efficiency gains, with the final SODNet realizing a 77.5% reduction in parameters and a 73.6% reduction in GFLOPs. Impressively, this optimization does not degrade core accuracy. The final model remains superior, achieving gains of 2.7% in precision, 1.2% in recall, and 1.2% in AP@50 compared to the baseline. These results demonstrate that SODNet successfully combines scale-aware accuracy improvements with a lightweight design, delivering a solution that achieves high accuracy and high efficiency for outfall detection.

**Table 2:** Model test results. YOLOv8: Baseline model; SODNet-O: Original unpruned model; SODNet: Final model.

Method	P$\uparrow$(%)	R$\uparrow$(%)	AP@50$\uparrow$(%)	Params$\downarrow$(M)	GFLOPs$\downarrow$	FPS$\uparrow$
YOLOv8	88.4	81.9	88.7	11.1	28.4	173.4
SODNet-O	91.3	84.2	90.1	6.6	19.0	177.7
SODNet	91.1	83.1	89.9	2.5	7.5	359.9

A particularly noteworthy finding is the significant gain in inference speed. SODNet reaches 359.9 FPS, meaning each image can be processed in just 2.78 ms. While SODNet-O already reduces computational load, its FPS improvement over the baseline is only 4.3 frames. In contrast, after channel pruning, the FPS jumps to 359.9, representing a 107.5% increase over the baseline. We attribute this to the core advantage of channel pruning as a structured simplification method. By directly reducing the width of feature maps, the network is compressed into a more compact and dense structure. Existing computation libraries can fully exploit this structure to achieve efficient inference without requiring specialized hardware or additional libraries. Structural simplification at the channel level also alleviates the critical hardware bottleneck of memory bandwidth, resulting in practical performance gains that far exceed the theoretical GFLOPs reduction ratio.

To provide a more detailed quantitative efficiency analysis, Table 3 presents a module-level breakdown of parameters and GFLOPs. The structural optimizations in the unpruned SODNet-O primarily reduce the computational burden in the neck and head. Specifically, the introduction of ECFPN and the MSGF-integrated decoupled head reduces GFLOPs in the neck and head by 3.4 and 6.0, respectively, compared to the baseline. Furthermore, the channel pruning strategy applied in the final SODNet significantly compresses the feature extraction stages, eliminating 3.1 M parameters from the backbone and 0.9 M from the neck. This module-level analysis confirms that our targeted architectural designs effectively mitigate computational redundancy.

**Table 3:** Module-level quantitative efficiency analysis of parameters and GFLOPs.

Method	Backbone		Neck		Head	
	Params$\downarrow$(M)	GFLOPs$\downarrow$	Params$\downarrow$(M)	GFLOPs$\downarrow$	Params$\downarrow$(M)	GFLOPs$\downarrow$
YOLOv8	5.1	12.5	3.9	7.8	2.1	8.1
SODNet-O	5.1	12.5	1.1	4.4	0.4	2.1
SODNet	2.0	5.1	0.2	1.2	0.3	1.2

### Ablation experiments

#### Effectiveness of the proposed modules

To analyze the contribution of each module to overall performance, we conducted ablation experiments on the iSOOD dataset. The results are presented in Table 4. The findings demonstrate that each module enhances SODNet’s performance.

**Table 4:** Results of ablation experiments.

YOLOv8	ECFPN	MSGF	MPDIoU	P$\uparrow$(%)	R$\uparrow$(%)	AP@50$\uparrow$(%)	Params$\downarrow$(M)	GFLOPs$\downarrow$	FPS$\uparrow$
$\checkmark$				88.4	81.9	88.7	11.1	28.4	173.4
$\checkmark$	$\checkmark$			91.1	82.7	89.1	7.5	23.7	170.6
$\checkmark$		$\checkmark$		89.8	82.9	88.6	11.6	25.1	167.1
$\checkmark$			$\checkmark$	89.6	81.7	88.9	11.1	28.4	**197.4**
$\checkmark$	$\checkmark$	$\checkmark$		**91.7**	82.5	89.8	6.6	19.0	173.8
$\checkmark$	$\checkmark$		$\checkmark$	91.3	83.8	89.6	7.5	23.7	161.9
$\checkmark$		$\checkmark$	$\checkmark$	90.3	82.4	89.3	11.6	25.1	164.3
$\checkmark$	$\checkmark$	$\checkmark$	$\checkmark$	91.3	**84.2**	**90.1**	**6.6**	**19.0**	177.7

First, incorporating the CAA module and the ACFF mechanism into ECFPN led to a marked improvement in feature extraction performance. Precision and recall increased by 2.7% and 0.8%, respectively, while parameters and computation decreased by 32.9% and by 16.5%. These results are consistent with our design objective of strengthening multi-scale feature representation through context awareness, thereby enabling more accurate target recognition in complex backgrounds.

Second, the introduction of the shared decoupled head reduced GFLOPs by 11.6% and increased recall by 1.0%, although the parameter count rose slightly by 4.5%. This suggests that the MSGF module enriched feature representation, making the model more sensitive to potential targets.

Third, adopting MPDIoU improved precision by 1.2% and significantly increased fps by 13.8%. By directly minimizing the distance between corner points, MPDIoU stabilizes the training process and accelerates convergence, thereby improving localization accuracy while speeding up inference.

It is noteworthy that integrating ECFPN and MSGF provided a dual advantage: it boosted detection accuracy and, unlike the individual modules, also decreased both the parameter size and computational cost. When explicitly comparing their individual impacts, the ECFPN contributed most significantly to the precision gains. The MSGF module was primarily responsible for reducing computational redundancy. Ultimately, when all three modules were integrated into the SODNet-O model, the best overall performance was achieved, with AP@50 of 90.1%, precision of 91.3%, and recall of 84.2%. These results surpassed those of any single module or partial combination, fully validating the synergy and complementarity of the proposed modules. Specifically, the model leverages ECFPN for efficient context-aware feature fusion, employs MSGF to improve the decoupled head’s interpretation of multi-scale features, and utilizes MPDIoU to ensure stable and accurate bounding box regression. Together, these modules establish an efficient multi-scale optimization framework tailored to the challenges of complex outfall detection tasks.

#### Comparison of feature fusion architectures

To validate the superiority of the ECFPN, a direct evaluation against existing advanced multi-scale modules was conducted under identical training settings. As presented in Table 5, replacing the standard FPN-PAN neck with BiFPN or HSFPN yields noticeable improvements in detection metrics while reducing the overall parameter count compared to the baseline. For instance, HSFPN improves precision to 89.7% and AP@50 to 89.3%. However, these alternatives still struggle to maximize target feature extraction while strictly minimizing computational overhead. In contrast, ECFPN achieves the highest precision and maintains the lowest computational cost, while keeping a highly competitive inference speed of 170.6 FPS. By actively filtering background noise and adaptively fusing contextual information, ECFPN strikes the optimal balance between multi-scale semantic enhancement and lightweight constraints, thereby firmly justifying its structural design for UAV deployment.

**Table 5:** Comparison of different feature fusion architectures on the iSOOD dataset.

Method	P$\uparrow$(%)	R$\uparrow$(%)	AP@50$\uparrow$(%)	Params$\downarrow$(M)	GFLOPs$\downarrow$	FPS$\uparrow$
Baseline (FPN-PAN)	88.4	81.9	88.7	11.1	28.4	**173.4**
BiFPN	88.8	82.6	88.8	7.4	25.0	169.7
HSFPN	89.7	82.4	**89.3**	**7.1**	23.9	172.3
ECFPN (ours)	**91.1**	**82.7**	89.1	7.5	**23.7**	170.6

### Hyperparameter sensitivity analysis

To evaluate SODNet’s robustness, we conducted a systematic sensitivity analysis of key hyperparameters. While keeping the foundational network configurations and standard training parameters consistent, we focused our quantitative evaluation on two structural factors that primarily influence the trade-off between detection precision and computational efficiency: the kernel configurations within the MSGF module and the pruning ratio threshold.

#### Sensitivity of MSGF kernel configurations

The MSGF module is primarily designed to enhance the extraction and fusion of multi-scale information. Table 6 summarizes the model’s sensitivity to different parallel convolution kernel combinations. Compared to a homogeneous kernel setting, such as using solely $3\times 3$ convolutions, the multi-scale combinations yield distinct performance shifts. The combination comprising $1\times 1$, $3\times 3$, and $5\times 5$ kernels significantly improves the recall to 83.8% but struggles to maximize precision. Ultimately, the configuration integrating $1\times 1$, $3\times 3$, $5\times 5$, and $7\times 7$ kernels achieves the optimal balance, delivering the highest precision of 89.8% and an AP@50 of 88.6%. Although this comprehensive receptive field introduces a marginal increase in parameters to 11.6 M and GFLOPs to 25.1, this configuration demonstrates the best overall performance among the tested variants for handling scale variations in UAV-based outfall images.

**Table 6:** Sensitivity analysis of different kernel configurations within the MSGF module.

Kernel configuration	P$\uparrow$(%)	R$\uparrow$(%)	AP@50$\uparrow$(%)	Params$\downarrow$(M)	GFLOPs$\downarrow$	FPS$\uparrow$
$\{3\times 3, 3\times 3, 3\times 3, 3\times 3\}$	89.3	80.5	88.3	**10.5**	**23.2**	161.6
$\{1\times 1, 3\times 3, 3\times 3, 5\times 5\}$	88.4	**83.8**	88.5	10.7	23.5	166.6
$\{1\times 1, 3\times 3, 5\times 5, 7\times 7\}$	**89.8**	82.9	**88.6**	11.6	25.1	**167.1**

#### Impact of pruning ratio threshold

Evaluating the pruning ratio (represented by the speed-up factor) is crucial for understanding the inherent trade-off between model compactness and representation capacity. As demonstrated in Table 7, varying the speed-up target from 1.5 to 3.5 significantly alters the network’s efficiency and accuracy. A conservative pruning target with a speed-up factor of 1.5 reduces parameters to 4.3 M while maintaining strong metrics. However, the optimal structural compression is achieved at a speed-up factor of 2.5. At this threshold, the model attains a peak precision of 91.1% and accelerates inference to 359.9 FPS, while simultaneously compressing parameters to merely 2.5 M and computation to 7.5 GFLOPs. Conversely, an excessively aggressive target with a speed-up factor of 3.5 results in severe feature degradation, leading to a drop in precision to 87.8% and a plummet in recall to 80.6%. Notably, this extreme sparsity also slightly degrades the inference speed to 345.3 FPS. These results justify selecting the 2.5 speed-up threshold as a suitable design choice for balancing efficiency and high accuracy.

**Table 7:** Sensitivity analysis of the pruning ratio (speed-up factor) on model performance and efficiency.

Pruning threshold	P$\uparrow$(%)	R$\uparrow$(%)	AP@50$\uparrow$(%)	Params$\downarrow$(M)	GFLOPs$\downarrow$	FPS$\uparrow$
SODNet-O	91.3	84.2	90.1	6.6	19.0	177.7
Speed-up 1.5	90.2	84.2	89.8	4.3	12.5	233.6
Speed-up 2.5	91.1	83.1	89.9	2.5	7.5	359.9
Speed-up 3.5	87.8	80.6	87.7	1.7	5.2	345.3

### Comparative experiments

To demonstrate the superiority of our method, SODNet was evaluated along with a diverse set of current object detection models. This set included the two-stage Faster R-CNN^23^, lightweight one-stage methods like RTMDet^46^, Hyper-YOLO^34^, and Mamba YOLO^47^, the Transformer-based RT-DETR^48^, and several modern YOLO models. Table 8 presents the results of this comparative study.

**Table 8:** Results of comparative experiments.

Method	P$\uparrow$(%)	R$\uparrow$(%)	AP@50$\uparrow$(%)	Params$\downarrow$(M)	GFLOPs$\downarrow$	FPS$\uparrow$
Faster R-CNN	90.5	82.7	88.8	41.3	90.9	38.4
RTMDet	85.4	80.6	86.9	4.9	8.0	83.6
YOLOv8	88.4	81.9	88.7	11.1	28.4	173.4
YOLOv10	88.6	83.5	89.5	8.0	21.6	175.4
YOLOv11	90.7	82.9	89.6	9.4	21.5	158.7
YOLO12	89.3	82.9	89.7	9.1	19.6	169.4
RT-DETR	90.3	**84.6**	88.6	19.9	56.9	85.4
Hyper-YOLO	88.4	83.5	89.4	3.9	10.8	206.3
Mamba YOLO	89.6	83.4	89.7	6.0	13.6	165.8
SODNet (ours)	**91.1**	83.1	**89.9**	**2.5**	**7.5**	**359.9**

SODNet outperforms all competing approaches in both detection accuracy and efficiency. Specifically, it achieves the highest AP@50 and precision among all comparison models. Although its recall is marginally lower than some methods, SODNet strikes a superior balance between precision and recall, resulting in a top-tier AP@50 score. In terms of efficiency, SODNet is also the most lightweight model evaluated. For instance, compared with the heavyweight Faster R-CNN, SODNet improves AP@50 by 1.1% while requiring only 5.9% of its parameters and 8.2% of its GFLOPs. This favorable accuracy-efficiency trade-off holds against other modern architectures. While SODNet’s recall is 1.5% lower than RT-DETR, it surpasses RT-DETR in precision and AP@50 by 0.8% and 1.3%, respectively, all while operating at a fraction of the computational cost. Similarly, compared to the state-space model Mamba YOLO, SODNet is 1.5% more precise, achieves a higher AP@50, and is also faster. Ultimately, SODNet achieves an inference speed of 359.9 FPS, a 74.4% improvement over Hyper-YOLO, the second-fastest model.

In conclusion, SODNet strikes an effective balance between high detection accuracy and low computational cost. Its combination of high precision, compact model size, and fast inference speed makes it highly promising for deployment on resource-constrained platforms, where it can effectively meet the demands of outfall detection tasks.

### Visualization analysis

**Fig. 9:**
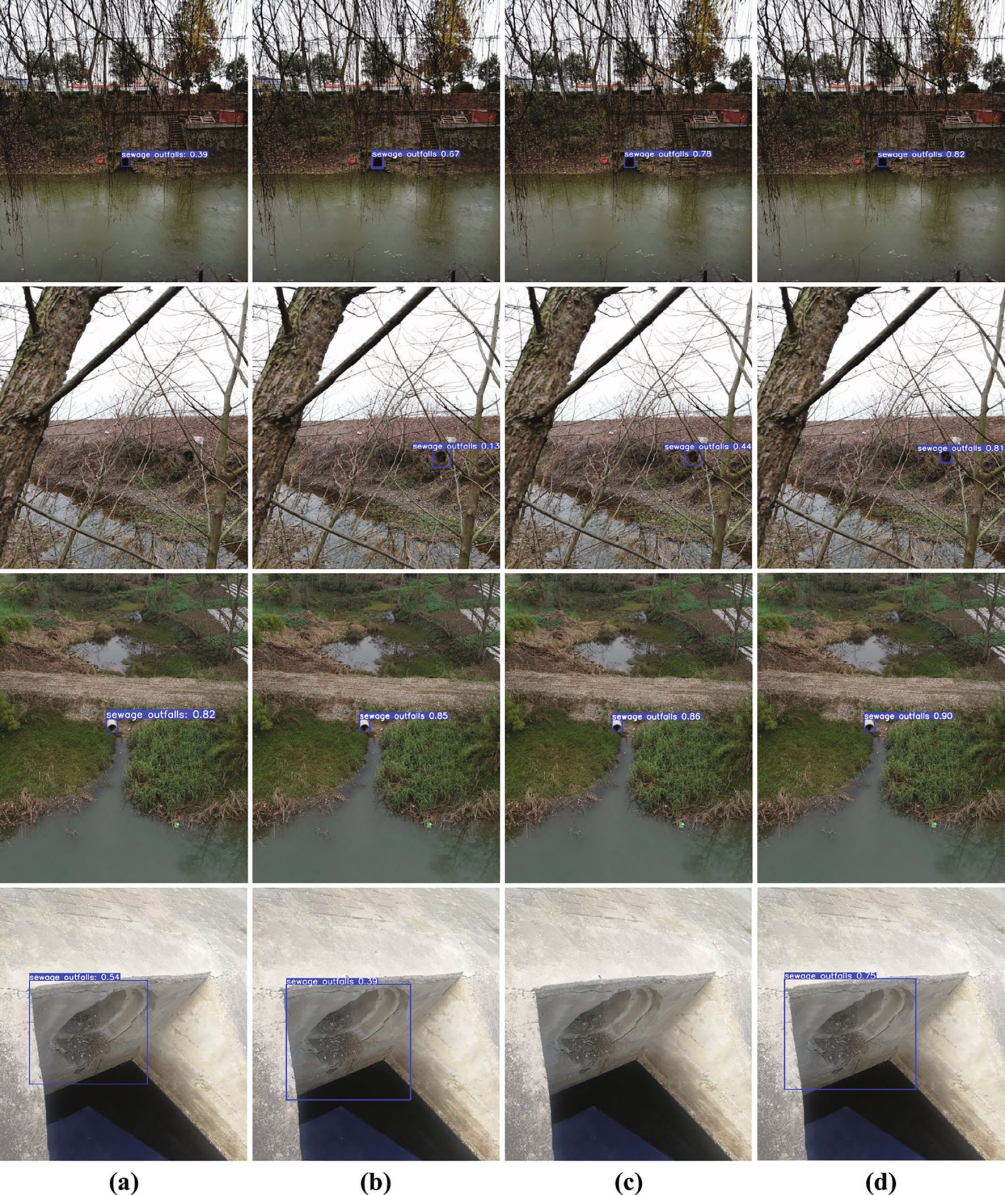
Detection results of different models. (**a**) Faster R-CNN; (**b**) YOLOv8; (**c**) YOLOv10; (**d**) SODNet.

To qualitatively assess the effectiveness and robustness of SODNet, we selected four representative images from the iSOOD dataset for intuitive evaluation. These images encompass challenges such as complex scenarios, multi-scale objects, and occlusion. The comparison models include the proposed SODNet, Faster R-CNN, YOLOv8, and YOLOv10. The results of the visual comparison are presented in Fig. 9. In complex natural scenes (second image), Faster R-CNN failed to detect the outfall effectively, and the detection results of YOLOv8 and YOLOv10 were also unsatisfactory. In contrast, SODNet accurately identified the target with high confidence. In scenarios with large-scale targets (the fourth image), SODNet also maintained stable detection performance. Overall, these results demonstrate that SODNet outperforms the comparison models in handling complex scenarios and multi-scale objects, while also preserving robustness in the presence of occlusion.

**Fig. 10:**
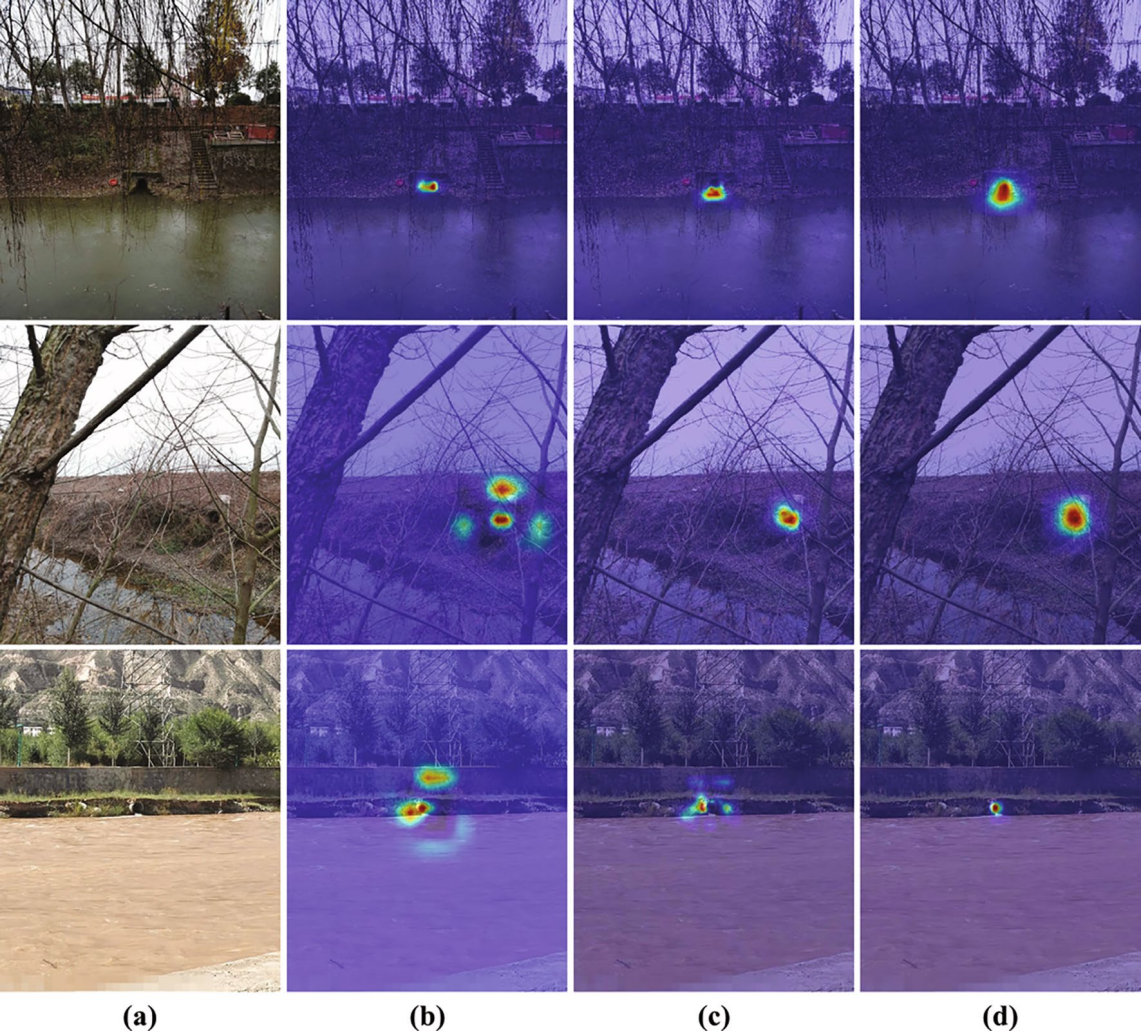
Grad-CAM heatmaps of different models. Red indicates high attention and greater contribution to prediction; blue indicates low attention and smaller contribution. (**a**) Original images; (**b**) YOLOv8; (**c**) YOLOv10; (**d**) SODNet.

To further explain the performance differences, gradient-weighted class activation mapping (Grad-CAM)^49^ was employed to generate heatmaps visualizing the attention regions of different models during detection, as shown in Fig. 10. YOLOv8 and YOLOv10 are more susceptible to background interference in complex scenarios, leading to attention shifts toward non-target regions and reduced accuracy. In contrast, SODNet focuses more on multi-scale objects, effectively mitigating background interference and achieving more stable detection performance.

### Deployment experiments

**Fig. 11:**
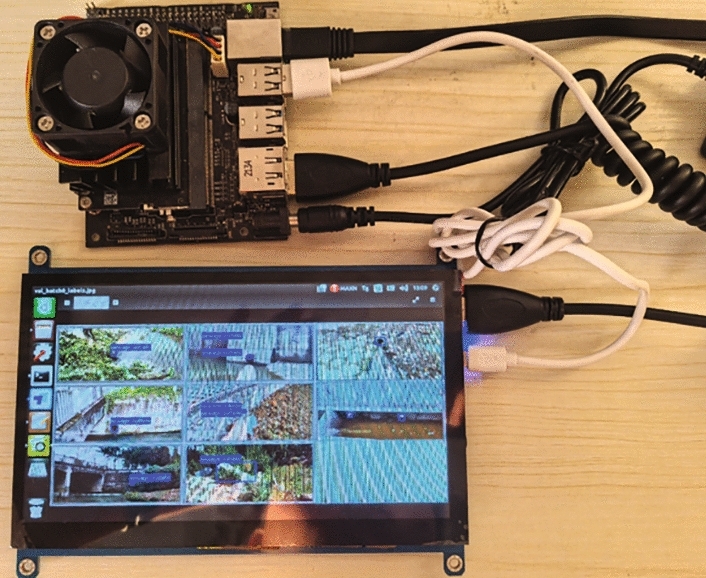
The actual deployment of SODNet on Jetson Xavier NX.

To ascertain if the promising efficiency exhibited by SODNet in a server environment translates to practical UAV applications, we deployed and evaluated the model on a resource-constrained edge device. The experimental platform was the NVIDIA Jetson Xavier NX (8GB), which integrates a Volta-architecture GPU with 384 CUDA cores and 48 Tensor cores, along with dual deep learning accelerators (NVDLA). Under the 15W power mode, the device delivers up to 21 TOPS of computational performance, making it an ideal platform for evaluating the inference efficiency of lightweight object detection models. No additional optimization techniques (e.g., TensorRT acceleration) were applied during the experiments. As shown in Fig. 11, SODNet was successfully deployed on this platform, demonstrating its feasibility in edge computing environments.

The experimental results indicate that SODNet achieves high efficiency on the Jetson Xavier NX. Table 9 compares SODNet with the baseline model YOLOv8. Compared with YOLOv8, SODNet improves FPS by 7.75%, reaching 40.3 FPS. Meanwhile, the model size and latency are reduced by 76.74% and 7.32%, respectively. These results confirm that SODNet outperforms YOLOv8 in inference speed, parameter efficiency, and model size, enabling fast, efficient operation on edge devices. This validation experiment provides a feasible technical solution for real-time sewage outfall detection using UAVs.

**Table 9:** Deployment performance results of YOLOv8 and SODNet on the Jetson Xavier NX platform.

Method	Params $\downarrow$ (M)	GFLOPs $\downarrow$	Model Size (MB) $\downarrow$	Latency (ms) $\downarrow$	FPS $\uparrow$
YOLOv8	11.1	28.4	21.5	26.77	37.4
SODNet	2.5	7.5	5	24.81	40.3

## Discussion

The performance advantages of SODNet stem primarily from its design strategy that balances scale-oriented accuracy with lightweight efficiency. By strengthening the contextual associations of multi-scale features, the model’s ability to distinguish targets from complex backgrounds is markedly enhanced, a conclusion further supported by Grad-CAM visualizations showing more focused attention distributions. More importantly, the channel pruning strategy yields inference speed gains far exceeding the theoretical reduction in computational load, reaching 359.9 FPS. In the deployment experiments, SODNet achieved 40.3 FPS on the Jetson Xavier NX.

In addition to these strengths, SODNet still leaves room for further exploration. One limitation to acknowledge is recall performance. While SODNet demonstrates high precision and competitive AP@50, its recall is slightly lower than that of some advanced detectors such as RT-DETR. This indicates that the model may occasionally miss smaller or more visually ambiguous outfalls. We attribute this slight reduction in recall directly to the aggressive channel pruning strategy. Specifically, to meet the strict real-time constraints of edge devices, the pruning process permanently removes a substantial number of feature channels. Although this effectively eliminates computational redundancy, it inevitably discards certain fine-grained feature representations that are highly sensitive to rare, small-scale, or heavily occluded targets. Nevertheless, considering the stringent hardware limitations of UAV platforms and the severe bottlenecks in air-to-ground data transmission (e.g., mmWave communication limitations and 3D mobility effects)^50^, this trade-off between a marginal drop in recall and a massive gain in inference speed is acceptable for practical deployment. Future research could explore adaptive pruning or hybrid feature aggregation mechanisms to mitigate this issue without compromising lightweight deployment.

Furthermore, the present study primarily focuses on the spatial localization of outfalls, whereas the core requirement of environmental monitoring is to assess their functional status and potential ecological impacts. Future research could extend SODNet by integrating its visual detection capabilities with multi-source data from UAV-mounted sensors such as thermal infrared^51^, hyperspectral imaging^52^, and geographic information^25^. For example, combining thermal infrared data could enable the identification of abnormal temperature signatures at outfalls, thereby determining whether they are actively discharging^53^. Through multimodal data fusion, SODNet could evolve beyond target detection toward functional assessment and impact analysis, advancing from static recognition to dynamic monitoring and intelligent early warning.

## Conclusion

This study proposes a lightweight, efficient deep learning–based detection model, SODNet, for precise identification of sewage outfalls in UAV imagery. The experimental evaluation reveals that SODNet excels at sewage outfall detection, achieving 91.1% precision and 89.9% AP@50. Notably, it also achieves a more efficient design, cutting parameter and computational cost by 77.5% and 73.6% respectively, relative to the baseline. Moreover, the deployed SODNet achieved 40.3 FPS on the Jetson Xavier NX. These results confirm that SODNet successfully unifies high accuracy, lightweight design, and real-time processing. Therefore, the proposed method provides an efficient tool for sewage outfall monitoring and management, with significant potential to mitigate water pollution and safeguard ecosystem stability and human health. Looking forward, a promising future perspective is to integrate the proposed UAV-based monitoring framework with satellite communication networks. Such advancements will overcome local communication barriers, thereby significantly enhancing the reliability and enabling large-area coverage for real-time environmental monitoring^54^. We hope that our research will further advance the integration of UAV remote sensing and water environmental monitoring.

## Data Availability

The dataset used in this study is publicly available at https://zenodo.org/records/10903574.
